# A Metagenomics Approach to Evaluate the Impact of Dietary Supplementation with *Ascophyllum nodosum* or *Laminaria digitata* on Rumen Function in Rusitec Fermenters

**DOI:** 10.3389/fmicb.2016.00299

**Published:** 2016-03-10

**Authors:** Alejandro Belanche, Eleanor Jones, Ifat Parveen, Charles J. Newbold

**Affiliations:** Institute of Biological, Environmental and Rural Sciences, Aberystwyth UniversityAberystwyth, UK

**Keywords:** *Ascophyllum nodosum*, brown seaweed, *Laminaria digitata*, phlorotannins, rumen microbiome, Rusitec

## Abstract

There is an increasing need to identify alternative feeds for livestock that do not compete with foods for humans. Seaweed might provide such a resource, but there is limited information available on its value as an animal feed. Here we use a multi-omics approach to investigate the value of two brown seaweeds, *Ascophyllum nodosum* (ASC) and *Laminaria digitata* (LAM), as alternative feeds for ruminants. These seaweeds were supplemented at 5% inclusion rate into a control diet (CON) in a rumen simulation fermenter. The seaweeds had no substantial effect on rumen fermentation, feed degradability or methane emissions. Concentrations of total bacteria, anaerobic fungi, biodiversity indices and abundances of the main bacterial and methanogen genera were also unaffected. However, species-specific effects of brown seaweed on the rumen function were noted: ASC promoted a substantial decrease in N degradability (−24%) due to its high phlorotannins content. Canonical correspondence analysis of the bacterial community revealed that low N availability led to a change in the structure of the bacterial community. ASC also decreased the concentration of *Escherichia coli* O157:H7 post-inoculation. In contrast, LAM which has a much lower phlorotannin content did not cause detrimental effects on N degradability nor modified the structure of the bacterial community in comparison to CON. This adaptation of the microbial community to LAM diets led to a greater microbial ability to digest xylan (+70%) and carboxy-methyl-cellulose (+41%). These differences among brown seaweeds resulted in greater microbial protein synthesis (+15%) and non-ammonia N flow (+11%) in LAM than in ASC diets and thus should led to a greater amino acid supply to the intestine of the animal. In conclusion, it was demonstrated that incorporation of brown seaweed into the diet can be considered as a suitable nutritional strategy for ruminants; however, special care must be taken with those seaweeds with high phlorotannin concentrations to prevent detrimental effects on N metabolism. This study highlights the value of combining fermentation and enzyme activity data with molecular characterization of the rumen microbiome in evaluating novel feeds for ruminants. Further experiments are required to determine the maximum seaweed inclusion rate tolerated by rumen microbes.

## Introduction

Seaweed is an important part of the coastal ecosystem, providing food and habitat for a variety of species. Excessive accumulation of seaweed on beaches and docks can however be harmful and is currently considered a public health hazard (Sheavly and Register, 2007). Seaweeds have traditionally been harvested and used as either fertilizers, due to their N and K content, or as thickening agents in food due to the high alginate concentration (Holdt and Kraan, 2011). Most of the carbohydrates and proteins in seaweed are however not digestible by humans or other monogastrics, thus the nutritional value of seaweed has traditionally been assumed to be in its contribution of minerals, trace elements and vitamins to the diet (Holdt and Kraan, 2011). A recent review describes the potential utilization of different seaweeds as dietary additives for various livestock species (Makkar et al., 2016), however their effect on rumen function is still largely unknown. In recent years it has been suggested that marine algae might be a rich source of structurally diverse bioactive compounds (Holdt and Kraan, 2011; Wijesekara et al., 2011). Seaweeds thrive in very exigent, competitive and aggressive environments which require the production of specific and potent active molecules, such as enzymes, peptides, fatty acids, antioxidants, tannins, lipids, and complex carbohydrates (Kim and Mendis, 2006).

Brown algae (Phaeophyta) are the only seaweeds which accumulate phlorotannins (PT) as an adaptive strategy of defense against stress conditions and herbivory (Li et al., 2011). PT are plant secondary metabolites based on phloroglucionol units (1,3,5-trihydroxybenzene) linked together through aryl-aryl, diaryl-ether or diaryl-diether bonds and biosynthesized by the acetate-malonate pathway (Singh and Bharate, 2006). Molecular sizes of PT range from 400 to 400,000 Da and their concentrations vary depending on the seaweed considered; it has been observed that the highest concentrations of PT appears in seaweed species which live at the mid-tide level, such as *Ascophyllum nodosum*, while those seaweed living on the low shore, such as *Laminaria digitata*, tend to have the lowest PT content (Connan et al., 2004).

Tannins contained in terrestrial plants (condensed and hydrolysable tannins) have been studied extensively in regard to their effects on ruminant nutrition (Mueller-Harvey, 2006). A reduction in protein degradation in the rumen is the most significant and well-known effect of terrestrial tannins (Min et al., 2003). On the contrary, there is little information about the effect of PT in ruminant nutrition. It has been reported that *A. nodosum* possess strong activity against ruminal microbes when incubated in batch cultures suggesting that this activity might be species-dependent (Wang et al., 2008). Moreover, the use of *Laminaria* as substrate to produce methane by anaerobic digestion has recently been investigated (Sutherland and Varela, 2014). In a previous experiment using batch cultures inoculated with rumen fluid, it was observed that *A. nodosum* was able to decrease protozoal activity and gas production rate while *L. digitata* increased VFA production when used at concentrations above 1 g/L (Belanche et al., 2015a). However, batch cultures are unable to sustain a stable fermentation over multiple days due to the accumulation of fermentation products. The rumen simulation technique (Rusitec) enables a long-term and stable *in vitro* fermentation to be maintained for several weeks, a time-scale sufficient to allow the study of the possible long-term adaptation of the rumen microbes that may occur (Patra and Saxena, 2009).

Therefore, this study investigates the activity and mode of action of two of the most common brown seaweeds when used as alternative feeds for ruminants: one with a high PT content (*A. nodosum*); and the second with low PT content (*L. digitata*). A multi-omics approach was adopted based on a detailed description of the rumen fermentation, enzymatic activity, microbial protein synthesis in addition to a thorough characterization of the bacterial and methanogens communities using Next Generation Sequencing.

## Materials and methods

### Diets and apparatus

Seaweeds (*A. nodosum* and *L. digitata*) were obtained from wild stock at afternoon low tide on a rock outcrop off Aberystwyth beach (52°42′N, 4°09′W, UK) on the 15th May 2014. Seaweed fronds were harvested at 5 cm above the soil level, frozen at −80°C within 1 h of harvesting, freeze-dried, ground to 1 mm^2^ particle size (A11 Basic IKA Mill, Staufen, Germany) and preserved at −20°C. A Rusitec rumen simulating fermentor was used to investigate the effect of three experimental diets consisting of a control diet alone (**CON**) or supplemented (5% inclusion rate in DM) with either *A. nodosum* (**ASC**) or *L. digitata* (**LAM**). Experimental diets had a 50:50 forage-to-concentrate ratio and all their ingredients were also ground to pass through 1 mm^2^ sieve size (Supplemental Table 1). The inclusion rate used in this experiment (equivalent to 1.56 g/L) was chosen based on the minimum effective dose observed in a previous dose-response study using rumen batch cultures (Belanche et al., 2015a).

Animal procedures were carried out in accordance with the Animal Scientific Procedures Act 1986 and protocols were approved by the Aberystwyth University Ethical Committee. Rumen fluid was obtained from four barren rumen-cannulated Holstein-Friesian cows fed at maintenance level (67% ryegrass hay and 33% concentrate, on a DM). Rumen contents were sampled before the morning feeding, filtered through a double layer of muslin and transferred to the lab at 39°C under anaerobic conditions. The trial consisted of a single incubation period using 12 vessels which were considered as experimental units. Each dietary treatment had four replicates which were randomly allocated to the vessels and inoculated with rumen fluid from different cows. Vessels had an effective volume of 800 ml and were kept at 39°C under permanent vertical agitation. On day 1 fermenters were inoculated with strained rumen fluid diluted 1:1 with artificial saliva (McDougall, 1948), then artificial saliva was continuously infused at a rate of 640 ml/d (dilution rate of 3.33%/h) to prevent the wash out of rumen microbes. Feed was placed in nylon bags (110 × 60 mm, pore size 100 μm^2^) and supplied to the vessels daily. Two feed bags remained in each fermenter at any time. The bag that had remained 2 days in each vessel was removed, squeezed and rinsed with 30 ml of artificial saliva. The liquid fraction of the washing was returned to the vessels and a new feed bag was inserted containing 20 g DM.

### Experimental procedure and sampling

The incubation trial was carried out over a period of 18 days (10 days for adaptation to the diet and 8 for sampling). On days 11, 12, 13, and 14, feed degradability, methane emissions and outflow of fermentation products were measured. Nylon bags were collected, rinsed with cold water for 20 min and degradability of nutrients after 48 h incubation was calculated from the loss in weight. Fermentation gasses were collected in hermetic bags to measure total gas and methane production by gas chromatography using an ATI Unicam 610 Series, Gas Chromatograph (UK). Production of fermentation products such as ammonia and VFA were measured from the overflow flasks which were kept on ice and with 10 mL of saturated HgCl_2_ (diluted 1:5) to stop the fermentation.

On days 15, 16, and 17 vessels fluids were sampled (15 mL) by aspiration at 2, 4, 8, and 24 h after feeding to describe the diurnal changes in the fermentation pattern. The pH was immediately recorded and each sample was divided into 4 subsamples: The 1^st^ subsample (10 mL) was snap frozen in liquid N for microbial characterization and enzymatic activity. The 2^nd^ subsample (1.6 mL) was diluted with 0.4 mL of deproteinising solution (20% orthophosporic acid containing 10 mM of 2-ethilbutyric acid) for VFA determination. The 3^rd^ subsample (0.8 mL) was diluted with 0.4 mL of trichloroacetate (25% wt/vol) for ammonia analysis, and the 4th subsample (0.8 mL) was snap frozen for lactate determination.

Microbial protein synthesis was measured using ^15^N as a microbial marker (Carro and Miller, 1999). On day 12 each fermenter was infused with 3 mg of ^15^N, as (^15^NH_4_)_2_SO_4_ to label the ammonia-N pool. From day 12 onwards ^15^N was added to the artificial saliva (3.7 mg/L) to label the microbial protein. On days 15, 16, and 17, residues of the feed bags were mixed with their respective effluents, homogenized in blender at low speed for 1 min and pooled to reconstitute the total digesta. One portion of total digesta (100 g) was frozen to generate the non-ammonia N (NAN) fraction, while the other portion (200 g) was used to measure ammonia-N and to isolate the total bacteria fraction (Belanche et al., 2013).

To evaluate the potential effect of ASC and LAM against pathogens, on the last day of the experiment (day 18) each vessel was inoculated with 5 mL of non-toxigenic *Escherichia coli* O157:H7 strain (NCTC 12900) grown for 2 days in liquid media (LB Broth, Thermo Fisher, UK). Following, this inoculation, serial samples (1 mL) were taken from the vessels at 0, 3, 6, 9, and 24 h to measure the concentration of *E. coli* plating 20 μL of sample on to Sorbitol MacConkey agar base N°3 (CM0813, Oxoid Ltd., Basingstoke, Hampshire, UK) containing rifampicin (50 μg/mL) and incubated at 37°C for 24 h prior to counting.

### Sample analyses

Feed chemical composition, methane production, microbial protein synthesis and concentration of protozoa, ammonia and VFA were determined as previously described (Belanche et al., 2013). Rumen protozoa were identified and quantified by optical microscopy (Dehority, 1993). Concentrations of total lactate and D-Lactate were measured using the Enzytec™ D/L-Lactic Acid kit (r-biopharm, Darmstadt, Germany) and L-lactate was calculated as the difference between them. Enzymatic activities in vessels content were measured using the colorimetric method previously described (Belanche et al., 2016).

### Seaweed analysis by HPLC-MS/MS

In order to identify the PT content in seaweeds, freeze-dried seaweed material was ground to a fine powder using a pestle and mortar and analyzed in triplicate by HPLC-MS/MS. Aqueous methanol (100 mL, 75% v/v) was added to the samples (10 g DM) and stirred for 5 h at ambient temperature. The solvent was decanted and the extraction procedure was repeated twice more with 75% aqueous methanol. The three solvent extracts were pooled and evaporated to 10 mL and stored at −20°C. The remaining seaweed residues were subjected to the same extraction procedure using 100% acetone (3 × 100 mL). Samples (10 mL) were centrifuged at 650 × g for 5 min and the supernatant was purified using a pre-cleaned reverse-phase cartridge (Sep-Pak 10 g C_18_ Waters, Hertfordshire, UK). The solvent was evaporated and the residue (1 mg) was re-suspended in methanol (1 mL) and centrifuged at 14,000 × g for 2 min. The supernatant (200 μL) was transferred into a HPLC vial. High performance liquid chromatography coupled with photodiode array detection (HPLC-PDA) was carried out as reported previously (Parveen et al., 2014). Samples (10 to 20 μL) were injected in a HPLC system fitted with a Waters C_18_ reverse-phase Nova-Pak column (4 μm, 8 mm, 100 mm, Waters Corporation, USA). The mobile phase consisted of acetate (95:5 v/v in water) and methanol (100%). Injection started with acetate alone followed by a linear increase in the proportion of methanol up to 75% in 70 min. The data were analyzed using Empower software (version 2002, Waters Corporation, USA).

Each sample (20 μL) was also subjected to high performance liquid chromatography coupled with photodiode array detection and electrospray ionization –tandem mas spectroscopy (HPLC/PDA/ESI/MS/MS) analysis (Parveen et al., 2011) using a LC/MS system (Thermo Electron Corporation, USA). The system comprised a Finnigan Surveyor PDA Plus detector and a Finnigan LTQ (linear trap quadrupole) with an ESI source. Chromatography was performed on a Waters C_18_ reverse-phase Nova-Pak column at a constant temperature of 30°C. The sample injection volume was 20 μL, the detection wavelength was set at 240–600 nm with a flow rate of 1 mL/min. Formate and methanol formed the mobile phase which varied from 95:5 v/v at the beginning to 0:100 v/v in 30 min. Instrumental set up were as follows; sheath gas 30 arbitrary units, auxiliary gas 15 units, spray voltage 4 kV, capillary temperature 320°C, capillary voltage 1.0V and tube lens offset 68V.

Volumes were recorded throughout all process to calculate total concentrations of phenols and phlorotannins in the samples. Quantifications were calculated using response factors. The abundances of phlorotannins, total phenols and carotenoids in the various extracts were determined by measurement of peak area in the HPLC chromatogram at 250 and 340 nm and reference to the extinction coefficient of chlorogenic acid. Soluble phenols were quantified using 5-caffeoylquinic acid (8.74 × 10^−7^μg/area unit) and expressed per unit of DM.

### DNA extraction and quantitative PCR (qPCR)

Lyophilized samples (100 mg DM) were bead-beaten for 1 min and DNA was extracted using a QIAamp DNA Stool Mini Kit (Qiagen Ltd., Crawley, UK) according to the manufacturer's instructions, but with the incubation temperature increased to 95°C for 10 min to maximize microbial lysis. All genomic DNA samples were further diluted (1/100) to decrease the concentration of PCR-inhibitors (i.e., proteins, polysaccharides or phenolic compounds) observed in ASC and LAM samples. Absolute concentration of DNA from total bacteria, protozoa, anaerobic fungi and methanogens were determined by qPCR and serial dilutions of their respective standards (10^−1^ to 10^−5^) as previously described (Belanche et al., 2015b). Briefly, qPCR was conducted in triplicate using a LightCycler® 480 System (Roche, Mannheim, Germany). Samples were prepared in 384-well plates using Epimotion 5075 Liquid Handling System (Ependorf®, Stevenage, UK). Amplification reaction (12.5 μL) contained DNA template (1 μL), 1 mM of each primer and 6.25 μl of SYBR Green JumpStart Taq ReadyMix (Sigma-Aldrich Ltd., Dorset, UK). Amplification conditions were 95°C for 5 min, then 25 cycles for bacteria (35 for methanogens) at annealing temperatures described in Supplemental Table 2 for 30 s, 72°C for 30 s and 95°C for 15 s, and a final melting analysis was performed to check primer specificity.

### Ion torrent next generation sequencing (NGS)

Rumen bacteria and methanogenic archaea communities were studied as previously described (de la Fuente et al., 2014). To improve sequencing depth only 1 sample per vessel was sequenced containing pooled DNA from the different time points. For bacterial profiling, amplification of the V1-V2 hypervariable regions of the 16S rRNA was carried out using bacterial primers (27F and 357R) followed by Ion Torrent adaptors (Supplemental Table 2). For methanogens profiling, amplification of the V2-V3 hypervariable region of the 16S rRNA was performed using archaeal primers (86F and 519R) also followed by adaptors (Supplemental Table 2). Forward primers were barcoded with 10 nucleotides to allow sample identification. PCR was conducted in duplicate; a 25 μL reaction was prepared containing DNA template (1 μl), 0.2 μM of each primer and 12.5 μL of KAPA HiFi Mix (Kapa Biosystems Ltd., London, UK). Amplification conditions for bacteria and methanogens were 95°C for 3 min, then 25 cycles of 98°C for 30 s, 58°C for 30s, 68°C for 45 s with a final extension at 68°C for 7 min. Resultant amplicons were visualized on a 1% agarose gel to assess quality of amplifications, then duplicate reactions were pooled. PCR products were further purified using Agencout AMpure XP beads (Beckman Coulter Inc., Fullerton, USA) and DNA concentration was assessed using an Epoch Microplate Spectrophotometer fitted with a Take3 Micro-Volume plate (BioTek, Potton, UK) to enable equi-molar pooling of samples with unique barcodes. Libraries were further purified using the E-Gel System with 2% agarose gel (Life Technologies Ltd., Paisley, UK). Purified libraries were assessed for quality and quantified on an Agilent 21000 Bioanalyzer with High Sensitivity DNA chip (Agilent Technologies Ltd., Stockport, UK). The emulsion PCRs were carried out using the Ion PGM Template OT2 200 Kit and the sequencing using the Ion Torrent Personal Genome Machine (PGM) system using the Ion PGM Sequencing 316™ Chip v2 for the bacterial library and the Ion PGM Sequencing 314™ Chip v2 for the methanogens library.

Following sequencing, sample identification numbers were assigned to multiplexed reads using the MOTHUR software environment. Data were de-noised by removing low quality sequences, sequencing errors and chimeras (quality parameters: maximum 10 homo-polymers, Q15 average over a 50 bp window, no mismatches allowed with barcode and 1 maximum with primer; Chimera check, both *de novo* and database driven using Uchime). Then, sequences were clustered into OTUS's at 97% identity using CD-HIT-OTU pipeline (http://weizhong-lab.ucsd.edu/cd-hit-otu/). The number of reads per sample were normalized to the sample with the lowest number of sequences. Bacterial taxonomic information on 16S rRNA sequences was obtained by comparing against the Ribosomal Database Project-II, while the methanogens were compared with the RIM-DB database (Seedorf et al., 2014). To exclude potential bacterial sequences from the methanogen dataset, methanogen sequences were blasted with the Ribosomal Database Project-II and those annotations which matched with bacterial sequences were removed. Only annotations with a bootstrap value over 80% were assigned, otherwise they were considered as unclassified. Raw sequence reads from the bacterial and methanogen libraries were deposited at the EBI Short Read Archive from the European Nucleotide Archive (accession numbers PRJEB11804 and PRJEB9814).

### Calculations and statistical analyses

Microbial N contribution to overflow was estimated based on the relationship between the ^15^N enrichment in the total digesta (NAN) and in the total bacterial-N pellet. The ability of bacteria to incorporate ammonia was calculated as the ratio between the ^15^N enrichments in the bacterial-N and in the ammonia-N fraction. Diet degradability, N metabolism and microbial diversity data were analyzed by ANOVA (Genstat 15th Edition, VSN International, UK) as follows:

$$\begin{array}{l} \text{Y}{}_{ijk}=\mu +D_{i}+A_{j}+e_{ijk} \end{array}$$

where *Y*_*ijk*_ is the dependent, continuous variable (*n* = 4), μ is the overall mean; *D*_*i*_ is the fixed effect of the diet (*i* = CON, ASC, LAM), *A*_*j*_ is the random effect of the animal inoculum (*j* = 1 to 4) and *e*_*ijk*_ is the residual error. For the rumen fermentation and qPCR data were analyzed using a repeated-measurements procedure (REML) including the different time-points after feeding (2, 4, 8, and 24 h). When significant effects were detected, treatment means were compared by Fisher's protected test. Findings with *P* < 0.05 were regarded statistically significant while *P* < 0.1 was considered as a tendency to differences.

Dietary effects on NGS log-transformed data were analyzed based on their Bray-Curtis distance metric within the function UPGMA. Data were then analyzed by non-parametric permutational multivariate analysis of variance using PRIMER-6 software (PRIMER-E Ltd., Plymouth, UK). Pairwise comparisons were also conducted to elucidate differences between treatments. The pseudo *F*-statistics and *P*-values were calculated after 999 random permutations of raw data using the Monte Carlo test. A Canonical Correspondence Analysis (CCA) was also performed to investigate the relationships between the structure of the bacterial and methanogens communities and the fermentation pattern. The significance of each variable was calculated using 999 random permutations. Bacterial and methanogens biodiversity indexes were calculated using normalized data to reduce over-inflation of true diversity in pyrosequencing data sets. For bacterial and methanogens relative abundances data were tested for normality and homogeneity using the Shapiro-Wilk and the Bartlett's tests, respectively, then data was log-transformed to attain normality.

## Results

### Seaweed composition

Chemical analysis of raw brown seaweeds revealed differences between ASC and LAM in terms of ash, N and carbon content (Table 1). HPLC analysis detected a cluster of phlorotannins which eluted between retention times 3–14 min in both seaweeds. Further MS/MS fragmentation of the pseudomolecular ions revealed that all of them had regular losses of 124 and 125 units (phloroglucinol). The major phlorotannin found had a retention time of 10.4 min and *m/z* 1037 in negative ion mode MS, while two smaller phlorotannin stereoisomers were detected at *m/z* 1861 and 1799. A fucoxanthin isomer was identified in positive ion mode MS with *m/z* 659, 641, 623, 581, 567, 563 and 549, respectively (Crupi et al., 2013). Raw extract of ASC had greater concentration of total phenols and phlorotannins than LAM, while the opposite was true for fucoxanthin concentrations (Table 1).

**Table 1:** **Chemical composition and concentration of phlorotannins in seaweeds (g/kg DM)**.

**Brown seaweed**	**ASC**	**LAM**
Ash	239	299
Nitrogen	18.7	26.7
Carbon	361	329
Total phenols	2.66	0.242
Phlorotannins	2.44	0.081
Fucoxanthin	0.116	0.587

### Fermentation pattern and microbial protein synthesis

Inclusion of 5% of ASC or LAM in the CON diet had no detrimental effects on feed degradation after 48 h-incubation in Rusitec (Table 2), but ASC decreased N degradability (*P* = 0.034). The amount of total gas, methane and metabolic hydrogen released and accepted during the fermentation process, as well as the individual VFA production were also unaffected by the experimental diets. ASC, in comparison to LAM, decreased the daily yield of NAN (*P* = 0.035) and microbial-N (*P* = 0.045). LAM tended (*P* = 0.075) to promote the highest efficiency of microbial protein synthesis (g microbial-N/kg DOM) and N utilization (g Microbial-N / kg N intake). Microbial N represented about 50% of the total NAN, whilst ammonia represented about 76% of the N uptake by the rumen bacteria for all diets.

**Table 2:** **Effect of 5% supplementation of a Control diet (CON) with *Ascophyllum nodosum* (ASC) or *Laminaria digitata* (LAM) on feed degradability, methanogenesis and microbial protein synthesis in the rumen simulating fermenter Rusitec**.

**Diets**	**CON**	**ASC**	**LAM**	**SED^1^**	***P*-value**
**DEGRADABILITY (%)**					
OM	68.6	63.7	68.5	2.58	0.176
N	60.0^a^	45.4^b^	56.2^a^	4.29	0.034
C	65.4	60.2	65.4	2.70	0.16
NDF	41.9	34.9	41.4	3.66	0.182
ADF	37.1	32.2	38.6	3.72	0.28
**GAS EMISSIONS**					
Total gas (L/d)	2.69	2.47	2.88	0.254	0.337
Methane (mM)	1.94	2.14	1.82	0.210	0.367
Methane (mmol/d)	5.24	5.36	5.23	0.864	0.986
Methane (mmol/gDOM)	0.40	0.44	0.40	0.057	0.733
[H] released^2^ (mmol/d)	108	103	112	11.35	0.753
[H] acepted^2^ (mmol/d)	106	100	108	10.81	0.777
[H] recovery^2^ (%)	97.6	96.8	96.2	1.99	0.774
**FERMENTATION PRODUCTS (mM/d**)					
Total VFA	63.3	59.1	65.7	5.94	0.561
Acetate	34.1	31.5	35.7	3.28	0.472
Propionate	13.0	13.9	14.9	2.46	0.755
Butyrate	6.81	6.64	6.46	0.857	0.919
**^15^N ENRICHMENT (%)**					
Ammonia	0.89	0.90	0.82	0.077	0.583
Bacteria	0.69	0.66	0.60	0.046	0.227
Digesta	0.34^a^	0.33^ab^	0.31^b^	0.009	0.045
**OUTFLOWS (mg/d)**					
Ammonia-N	117	168	166	36.80	0.355
NAN	456^ab^	422^b^	469^a^	13.84	0.035
NANM-N^3^	227	209	223	16.32	0.545
Microbial-N	228^ab^	213^b^	246^a^	10.02	0.045
**EMPS**					
Microbial-N: NAN	0.50	0.50	0.52	0.025	0.658
Microbial-N: N intake	0.55^ab^	0.51^b^	0.58^a^	0.024	0.075
Microbial-N from ammonia	0.79	0.74	0.75	0.115	0.874
Microbial-N/DOM (mg/g)	17.2^b^	17.5^ab^	18.9^a^	0.62	0.075

1 *Within a raw means without a common superscript differ (P < 0.05, n = 4)*.

2 *Metabolic hydrogen stoichiometric calculated based on VFA production (Ungerfeld, 2015)*.

3 *NANM-N; non-ammonia non-microbial N calculated by subtracting microbial N from non-ammonia N flow*.

**Figure 1:**
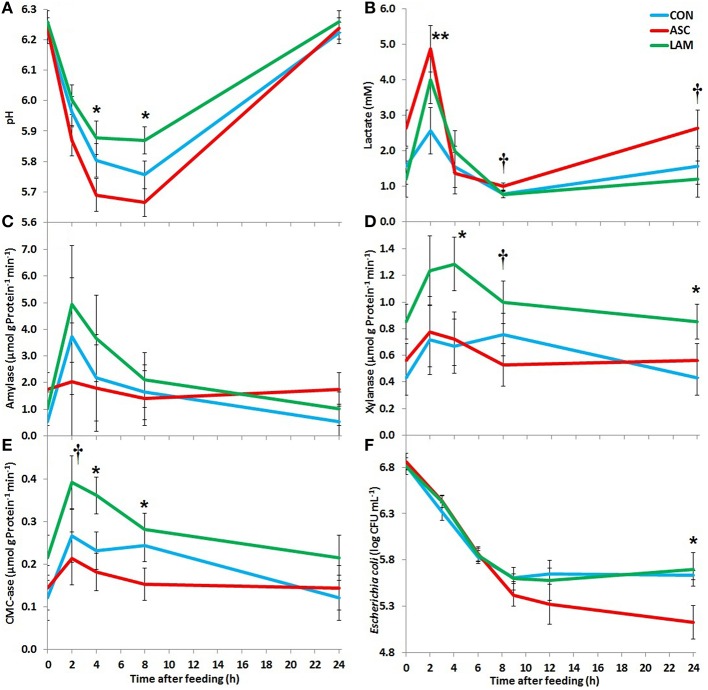
**Effect of 5% supplementation of a Control diet (CON) with *Ascophyllum nodosum* (ASC) or *Laminaria digitata* (LAM) on pH (A), lactate concentration (B), amylase activity (C), xylanase activity (D), carboxymethyl-cellulase activity (E), and *Escherichia coli* concentrations (F) post-inoculation in the rumen simulating fermenter Rusitec**. Error bars indicate the standard error of the difference for each time point (*n* = 4). ^**^P < 0.01, ^*^P < 0.05, ^†^*P* < 0.1.

Significant diurnal changes in fermentation pattern were observed (Table 3) in terms of pH, VFA, ammonia and lactate concentrations. The peak of fermentation occurred at 2 h after feeding for amylase, xylanase and CMC-ase absolute and relative enzymatic activities (Table 4). Fermentation pattern was not substantially affected by the experimental treatments in terms of pH, ammonia, lactate and VFA concentrations. ASC increased the molar proportion of iso-valerate (*P* < 0.001) with a decrease in valerate (*P* = 0.001), while LAM increased the D/L lactate ratio (*P* = 0.045). A significant interaction between diet and time was observed for vessels pH (*P* = 0.005) indicating that ASC was the only treatment able to buffer and delay the post-prandial drop in pH (Figure 1). In terms of absolute enzymatic activity (μ mol of sugar released /g DM × min) no differences were observed in amylase and xylanase activity, while ASC caused a decrease in the CMC-ase activity compared to CON and LAM (*P* = 0.007). A greater concentration of enzymes (as protein/gDM) was observed in CON than LAM vessels (*P* = 0.016). As a result, the LAM diet promoted a greater xylanase (*P* = 0.024) and CMC-ase (*P* = 0.005) relative activity (expressed as μmol of sugar released /g Protein × min) in comparison to CON and ASC diets, while amylase relative activity was unaffected by diets. Figure 1 showed that these difference in the enzymatic activity mainly occurred during the mid-fermentation period (4–8 h after feeding).

**Table 3:** **Effect of 5% supplementation of a Control diet (CON) with *Ascophyllum nodosum* (ASC) or *Laminaria digitata* (LAM) and the sampling time on rumen fermentation parameters in the rumen simulating fermenter Rusitec**.

	**Diets**			**SED^1^**	***P*-value**	**Time after feeding**				**SED^2^**	***P*****-value**	
	**CON**	**ASC**	**LAM**		**Diet**	**2 h**	**4 h**	**8 h**	**24 h**		**Time**	**Diet × Time**
pH	5.94	5.95	5.92	0.054	0.808	5.98^B^	5.81^C^	5.78^C^	6.17^A^	0.015	<0.001	0.005
Ammonia-N (mg/dL)	14.4	12.5	16.0	6.610	0.873	21.9^A^	8.26^B^	3.99^B^	23.1^A^	3.690	0.002	0.626
Total VFA (mM)	135	141	141	3.260	0.182	132^B^	145^A^	150^A^	129^C^	3.450	<0.001	0.561
**MOLAR PROPORTION**												
Acetate	54.1	54.3	55.0	0.968	0.621	55.1^A^	54.8^A^	54.8^A^	53.1^B^	0.347	<0.001	0.122
Propionate	24.7	23.3	22.6	2.974	0.777	22.9^B^	23.4^B^	23.2^B^	24.7^A^	0.302	<0.001	0.128
Butyrate	10.3	13.5	11.3	1.507	0.182	11.9	11.7	11.6	11.6	0.196	0.437	0.208
Iso-butyrate	0.73	0.73	0.65	0.041	0.155	0.69^B^	0.65^C^	0.68^BC^	0.80^A^	0.016	<0.001	0.026
Valerate	4.66^a^	3.24^b^	4.50^a^	0.218	0.001	4.03	4.19	4.14	4.17	0.128	0.532	0.896
Iso-valerate	1.64^c^	2.65^a^	2.27^b^	0.127	<0.001	2.19^B^	2.02^C^	2.14^BC^	2.38^A^	0.068	0.008	0.627
Caproate	3.02	1.97	2.92	0.787	0.394	2.66^A^	2.67^A^	2.73^A^	2.49^B^	0.068	0.041	0.338
**LACTATE (mM)**												
Total	1.61	1.77	2.19	0.307	0.229	3.21^A^	1.64^B^	0.82^C^	1.75^B^	0.314	<0.001	0.360
D-lactate	0.83	0.92	1.10	0.117	0.146	1.23^A^	0.71^B^	0.34^C^	1.52^A^	0.141	<0.001	0.607
L-lactate	0.78	0.85	1.09	0.221	0.395	1.98^A^	0.93^B^	0.48^CB^	0.23^C^	0.195	<0.001	0.270
Ratio D/L	2.00^b^	2.13^ab^	2.41^a^	0.126	0.045	0.65^B^	0.78^B^	0.71^B^	6.58^A^	0.186	<0.001	0.073

1 *Standard error of the difference for the effect of the diet (average of all time points, n = 16)*.

2 *Standard error of the difference for the effect of the time (average of all diets, n = 12). Within a raw means without a common superscript differ among diets (lowercase) or time-points (uppercase), P < 0.05*.

**Table 4:** **Effect of 5% supplementation of a Control diet (CON) with *Ascophyllum nodosum* (ASC) or *Laminaria digitata* (LAM) and the sampling time on rumen enzymatic activity and microbial numbers in the rumen simulating fermenter Rusitec**.

	**Diets**			**SED^1^**	***P*-value**	**Time after feeding**				**SED^2^**	***P*****-value**	
	**CON**	**ASC**	**LAM**		**Diet**	**2 h**	**4 h**	**8 h**	**24 h**		**Time**	**Diet × Time**
Protein (mg/gDM)	11.8^a^	10.2^ab^	9.52^b^	0.567	0.016	10.6	9.81	10.2	11.6	1.043	0.362	0.654
**ABSOLUTE ENZYMATIC ACTIVITY**^3^												
Amylase	0.95	0.54	0.71	0.328	0.506	1.12^A^	0.84^AB^	0.56^BC^	0.42^C^	0.143	0.003	0.082
Xylanase	0.26	0.21	0.35	0.056	0.115	0.31^A^	0.28^AB^	0.24^C^	0.25^BC^	0.014	0.004	0.210
Carboxymetyl-cellulase	0.09^a^	0.06^b^	0.10^a^	0.009	0.007	0.10^A^	0.09^B^	0.07^BC^	0.06^C^	0.008	<0.001	0.151
**RELATIVE ENZYMATIC ACTIVITY**^3^												
Amylase	2.03	1.74	2.93	1.260	0.638	3.58^A^	2.54^AB^	1.72^B^	1.10^B^	0.580	0.025	0.304
Xylanase	0.64^b^	0.65^b^	1.09^a^	0.135	0.024	0.91^A^	0.89^A^	0.76^AB^	0.61^B^	0.101	0.041	0.618
Carboxymetyl-cellulase	0.22^b^	0.17^b^	0.31^a^	0.026	0.005	0.29^A^	0.26^AB^	0.23^B^	0.16^C^	0.028	0.003	0.464
**MICROBIAL NUMBERS (LOG)**												
Bacteria (pg/gDM)	6.18	6.17	6.22	0.062	0.714	6.05	6.27	6.23	6.21	0.105	0.215	0.500
Methanogens (copies/gDM)	9.94^a^	8.88^c^	9.34^b^	0.112	<0.001	9.15	9.46	9.40	9.55	0.161	0.142	0.377
Methanogens (10^3^×ΔC_T_)	3.27^a^	0.33^b^	0.72^b^	0.458	0.001	0.93	1.51	1.10	2.22	0.560	0.170	0.216
Anaerobic fungi (10^8^ × pg/gDM)	2.12	4.16	1.91	1.247	0.219	2.50	3.29	0.94	4.20	1.297	0.146	0.315
Protozoa (pg/gDM)	4.14^a^	1.30^b^	1.42^b^	0.507	0.002	2.01	2.22	2.67	2.24	0.255	0.126	0.120

1 *Standard error of the difference for the effect of the diet (average of all time points, n = 16)*.

2 *Standard error of the difference for the effect of the time (average of all diets, n = 12). Within a raw means without a common superscript differ among diets (lowercase) or time-points (uppercase), P < 0.05*.

3 *Absolute and relative enzymatic activity were expressed in: (μmol of sugar / gDM × min) and (μmol of sugar / g Protein × min), respectively*.

Quantitative PCR revealed no diurnal changes in the absolute concentration of bacterial, methanogen, fungal or protozoal DNA. Similarly, no differences across diets were observed in the bacterial or fungal DNA concentrations. In comparison to CON, seaweed diets had a lower concentration of protozoal DNA (*P* = 0.002), in addition to lower methanogen levels in absolute numbers (*P* < 0.001) and in relative numbers with respect to total bacteria (*P* = 0.001). This decrease in protozoal DNA was however not associated with a decrease in total protozoal numbers or the abundance of the different protozoal groups.

### Bacterial 16S rDNA sequencing

**Figure 2:**
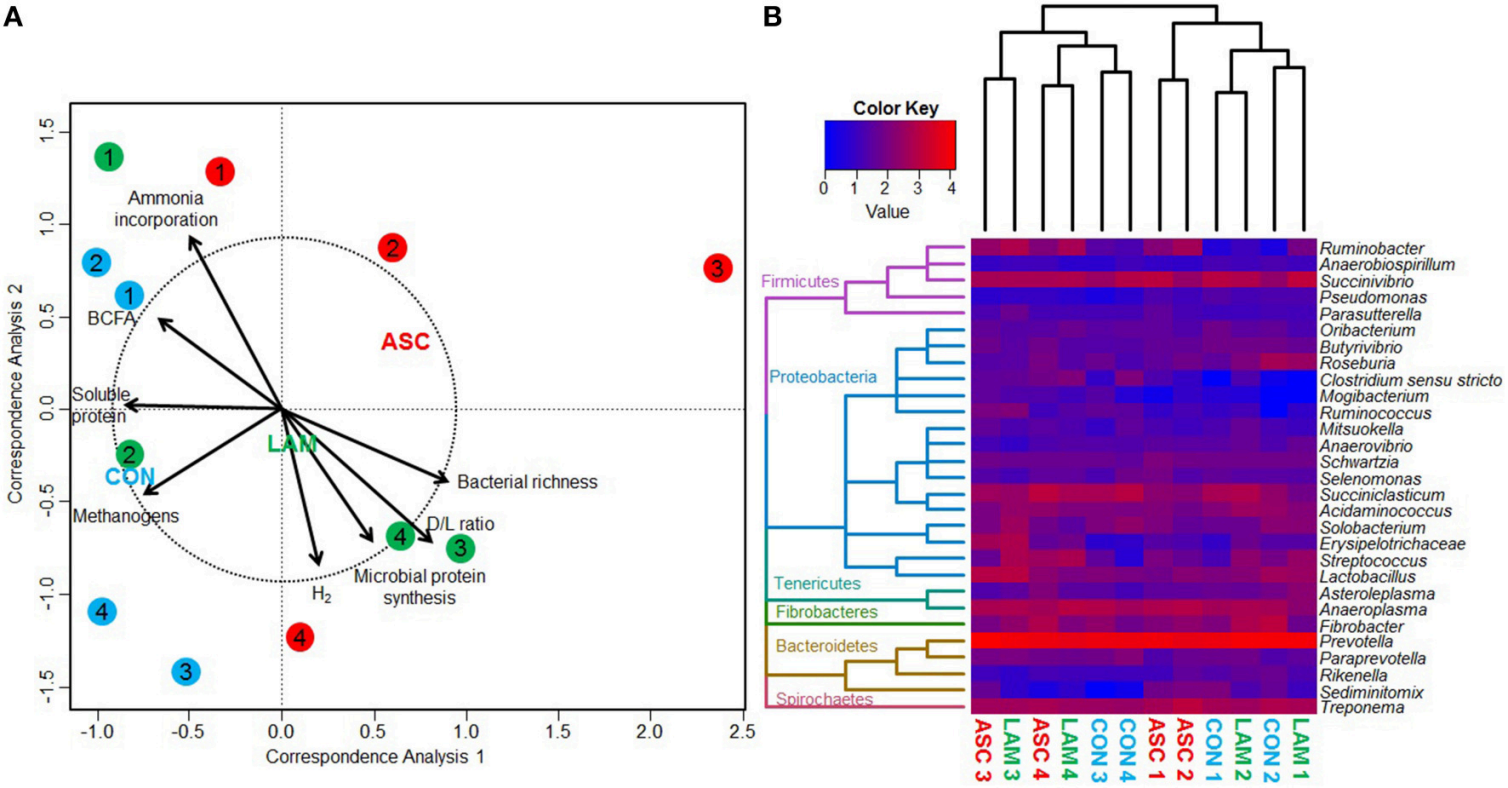
**(A)** Canonical correspondence analysis illustrating the relationship between the structure of the bacterial community and the rumen fermentation pattern in the rumen simulating fermenter Rusitec. Arrows show the direction of the gradient and those longer that the dotted circle are significant (*P* < 0.05). Centroid is indicated for each treatment: Control (CON), *Ascophyllum nodosum* (ASC) and *Laminaria digitata* (LAM). Animals used as donors are indicated in numbers. **(B)** Heat map describing the effect of the diet on the structure of the bacterial comunity and on the abundance of the main genera. Dendrogram is based on the UPGMA clustering of the Bray-Curtis distances. The total number of reads per sample was log transformed and minor genera discarded (<0.1%).

Bacterial 16S rDNA sequencing generated 2.1 million raw sequences. Quality filtering resulted in 578,496 high quality sequences (average length 285 bp) that were clustered in to 1061 unique OTU's with 24,655 sequences per sample after normalization. Permutational analysis of variance (Table 5) showed a moderate effect of the experimental diet on the structure of the bacterial community (*P* = 0.052). Pair-wise analysis showed that the structure of the bacterial community with ASC supplementation differed to that observed in CON diets (*P* = 0.048), while no differences were observed between ASC and LAM or between CON and LAM. In order to study the factors which correlate with the structure of the bacterial community, a Canonical Correspondence Analysis (CCA) was performed (Figure 2A). This analysis showed a separation between ASC and CON samples in the ordination plot (horizontal axis) while LAM samples were placed between COM and ASC. Moreover, several variables were correlated with the sample distribution: concentration of soluble protein (*P* = 0.091), methanogens (*P* = 0.082) and branched chain fatty acids (*P* = 0.092), as well as the ammonia incorporation by bacteria (*P* = 0.017) tended to positively correlate with the structure of the bacterial community in CON samples (left size) and negatively correlated with ASC samples (right side). Other variables such as D/L lactate ratio (*P* = 0.017), bacterial richness (*P* = 0.046), metabolic hydrogen production (*P* = 0.067) and microbial protein synthesis (*P* = 0.073) also tended to correlate with the structure of the bacterial community but this effect was not clearly associated to any experimental treatment.

**Table 5:** **Effect of 5% supplementation of a Control diet (CON) with *Ascophyllum nodosum* (ASC) or *Laminaria digitata* (LAM) on the structure of the bacterial and methanogens communities in the rumen simulating fermenter Rusitec**.

**Community^1^**	**Bacteria**			**Methanogens**		
	**Similarity**	**Pseudo-F**	***P*-value**	**Similarity**	**Pseudo-F**	***P*-value**
Treatment effect		1.91	0.052		0.68	0.694
**PAIRWISE COMPARISONS**						
CON vs. ASC	68.8	1.74	0.048	76.9	1.05	0.408
CON vs. LAM	70.8	1.28	0.202	76.7	0.26	0.974
ASC vs. LAM	71.4	1.07	0.413	78.7	0.97	0.438

1 *Higher Pseudo-F and lower similarities and P-values correspond to greater differences in the microbial composition (n = 4)*.

In terms of bacterial diversity, no differences across diets were observed for Chao index and Good's coverage, indicating that the sequencing depth was comparable across treatments (Table 6). Similarly no differences were observed in any of the diversity indexes which remained high and constant indicating the presence of a highly diverse bacterial community in which most of the bacterial species had similar abundance across diets.

**Table 6:** **Effect of 5% supplementation of a Control diet (CON) with *Ascophyllum nodosum* (ASC) or *Laminaria digitata* (LAM) on the structure of the bacterial, methanogen and protozoal communities in the rumen simulating fermenter Rusitec**.

**Diets^1^**	**CON**	**ASC**	**LAM**	**SED**	***P*-value**
**BACTERIA**					
Richness	590	703	613	47	0.114
Shannon	4.76	4.72	4.7	0.222	0.965
Evenness	0.75	0.72	0.73	0.028	0.669
Simpson	0.98	0.96	0.97	0.017	0.622
Chao	859	891	729	119.0	0.407
Good's	0.75	0.76	0.80	0.047	0.622
**METHANOGEN**					
Richness	8.25	8.25	9.00	0.935	0.67
Shannon	0.77	0.63	0.84	0.214	0.647
Evenness	0.35	0.31	0.38	0.094	0.718
Simpson	0.39	0.31	0.45	0.139	0.663
Chao	8.25	8.50	9.38	0.977	0.770
Good's	0.91	0.86	0.84	0.050	0.65
**PROTOZOA**					
Total (log cells/mL)	2.41	2.80	2.53	0.272	0.400
Subf. Entodiniinae (%)	90.3	86.4	92.2	4.140	0.415
Epidininum (%)	0.00	1.23	2.78	2.219	0.497
Subf. Diplodiniinae (%)	8.39	12.4	3.55	4.390	0.213
Holotrichs (%)	1.25	0.00	1.47	1.701	0.666

Based on the classification by RDPII, Bacteroidetes was the most abundant phylum across diets (46%), followed by Firmicutes (22%), Proteobacteria (4.4%), Tenericutes (2.8%), Spirochaetes (2.2%), Fibrobacteres (1.4%), minor phyla (0.1%), whereas some sequences were unclassified (22%). No substantial differences between diets were observed at the phylum level (Figure 2B and Supplemental Table 3). ASC and LAM tended to increase the abundance of *Proteobacteria* (*P* = 0.061) in comparison to CON, with *Ruminobacter* the genus showing the greatest response (*P* = 0.019). Within the phylum *Bacteroidetes* LAM increased the abundance of the family *Flammeovirgaceae* (*P* = 0.007). Within the plylum *Firmicutes;* LAM increased the abundance of *Streptococcus* with respect to CON (*P* = 0.034), while ASC increased the abundance of *Pseudobutyrivibrio* with respect to LAM (*P* = 0.045).

LAM had no specific anti-microbial effect against *E. coli* since post-inoculation concentrations in the vessels followed similar decay pattern to that observed in CON (Figure 1F). On the contrary, *E. coli* numbers decreased to significantly lower levels 24 h post-inoculation (*P* = 0.038) in ASC supplemented vessels.

### Methanogens 16S rDNA sequencing

**Figure 3:**
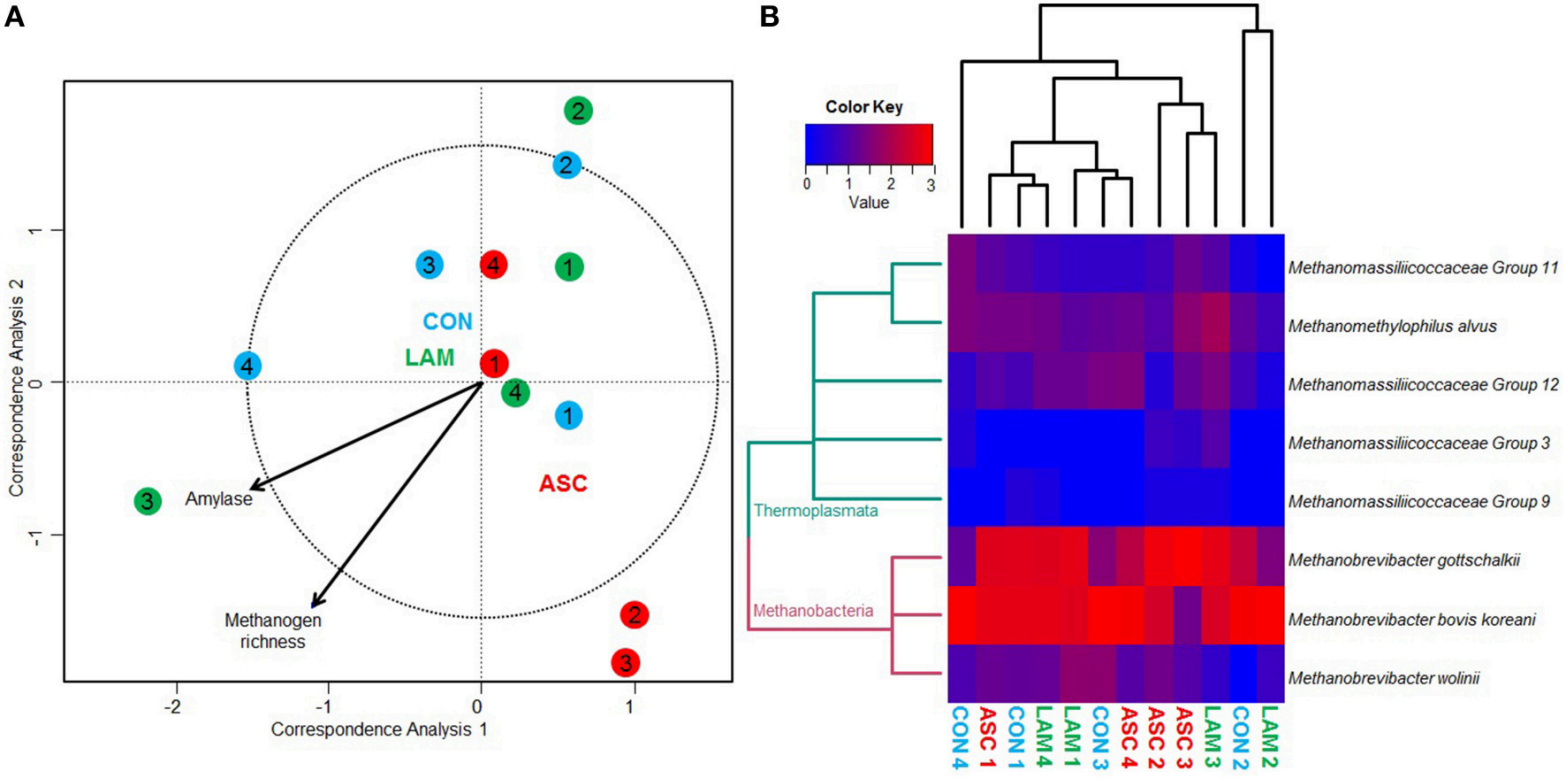
**(A)** Canonical correspondence analysis illustrating the relationship between the structure of the methanogen community and the rumen fermentation pattern in the rumen simulating fermenter Rusitec. Arrows show the direction of the gradient and those longer that the dotted circle are significant (*P* < 0.05). Centroid is indicated for each treatment: Control (CON), *Ascophyllum nodosum* (ASC), and *Laminaria digitata* (LAM). Animals used as donors are indicated in numbers. **(B)** Heat map describing the effect of the diet on the structure of the methanogen community and on the abundance of the main methanogen species. Dendrogram is based on the UPGMA clustering of the Bray-Curtis distances. The total number of reads per sample was log transformed and minor genera were discarded (<0.1%).

Methanogens sequencing generated a 0.29 million raw sequences. Quality filtering resulted in 48,405 high quality sequences (average length 380 bp) that were clustered in to 12 unique OTU's with 1001 sequences per sample after normalization. Permutational analysis of variance revealed that the structure of the methanogen population was stable and unaffected by the diet or the inoculum used (Table 5). As a result, pair-wise analysis showed no significant differences between the diets studied. Similarly, CCA showed no clear separation of the samples in the ordination plot according to the experimental treatments (Figure 3A). Moreover, the structure of the methanogen community was not correlated with most of the fermentation parameters. Methanogen richness (*P* = 0.019) and amylase enzymatic activity (*P* = 0.034) were correlated with the structure of this community but this was not driven by the experimental diet.

**Figure 4:**
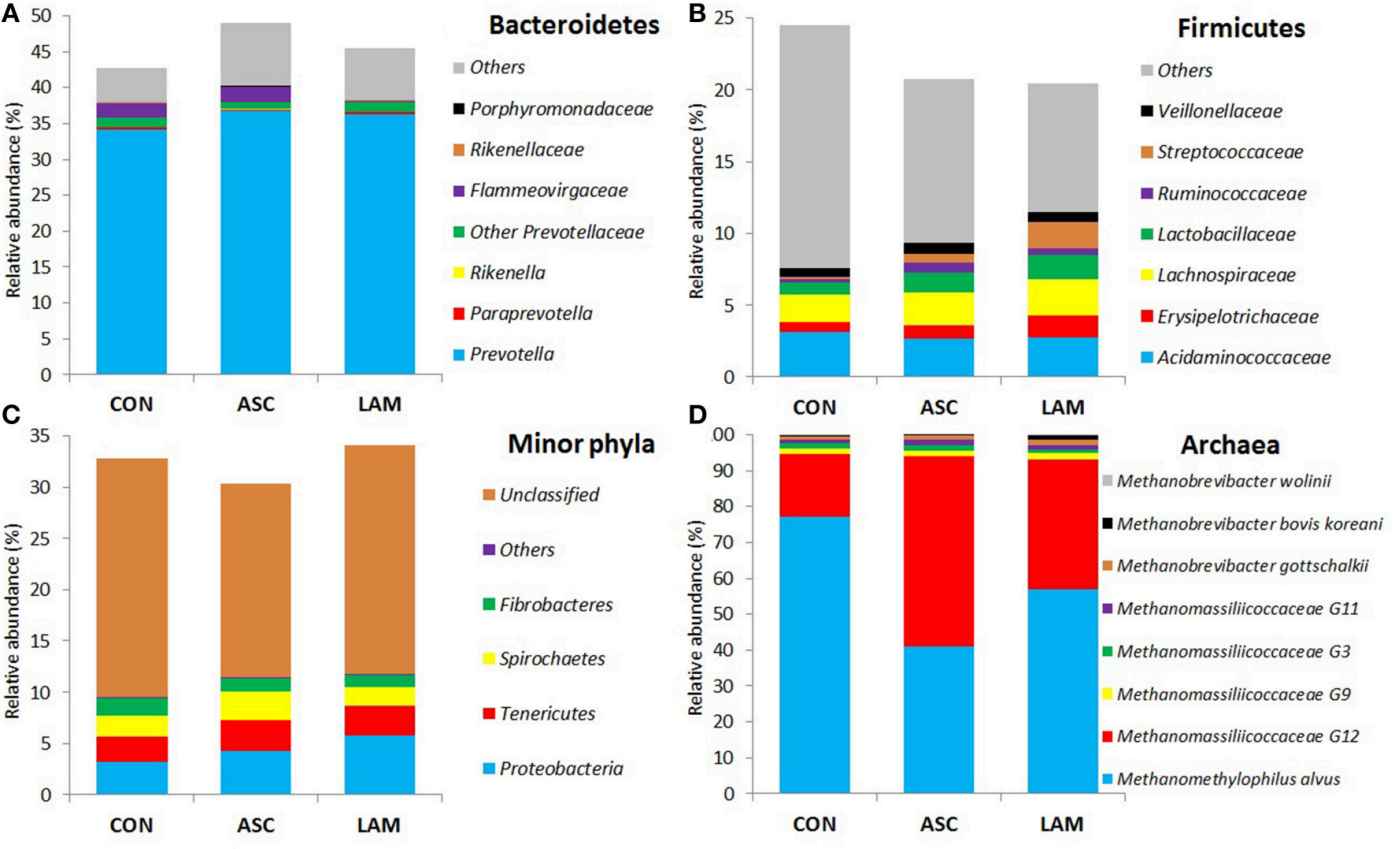
**Effect of 5% supplementation of a Control diet (CON) with *Ascophyllum nodosum* (ASC) or *Laminaria digitata* (LAM) on abundance of the main bacteria (A–C) and methanogen (D) phyla and families in the rumen simulating fermenter Rusitec**.

Similar to bacteria, methanogen 16S rDNA sequencing showed no differences across diets in Chao index and Good's coverage, indicating a homogeneous sequencing depth across treatments (Table 6). Moreover, diversity indices of the methanogen community were unaffected by the experimental diet. Based on the RIM-DB database, only two families comprised of the entire methanogens population (Figure 4): Methanomassiliicoccaceae (class Thermoplasmata) and Methanobacteriaceae (class Methanobacteria). Supplementation of CON diet with ASC or LAM did not modify the abundance of the methanogen groups due to their high variation within treatments (Figure 3B and Supplemental Table 4).

## Discussion

### Feed degradability and methanogenesis

*Ascophyllum nodosum* is one of the most common brown seaweeds growing in the North Atlantic Ocean. This seaweed is very dark due to a high content of phenolic compounds. In this study the majority of the phenols (91%) observed in ASC corresponded to phlorotannins as they showed a pattern of losses of phloroglucinol units in the HPLC/MS/MS analysis (Steevensz et al., 2012; Wang et al., 2012). As a result, ASC contained around 30 folds more total phenols and phlorotannins than observed in LAM. This observation agrees with previous findings which revealed between 23 and 70 times greater phlorotannin content in ASC than LAM depending on the season or the quantification method considered (Connan et al., 2004; Steevensz et al., 2012). Thus, it is likely that most of ASC protein (11.7% protein content in our experiment) would be bound to phenols through oxidative and nucleophilic reactions protein-phenol complexes which are not attacked by bacteria (Wang et al., 2008). This lack of protein degradability is a distinct drawback to *Ascophyllum* meal (Indergaard and Minsaas, 1991). In our experiment *Ascophyllum* represented 5% of the total N in the ASC diet but promoted a decrease of 15 percentage units in the N degradability in comparison to the CON diet. This observation suggest that the main effect of ASC on protein degradability does not correspond to its fully indigestible protein content, but to the inhibition of the feed protein degradability as a result of the formation of PT-protein complexes with other proteins. A similar mode of action based on the formation of reversible bounds with proteins and carbohydrates has been described for terrestrial tannins (Min et al., 2003) promoting a negative impact on protein (−3.7%), OM (−2.6%), and fiber (−2.9%) degradability in ruminant diets (Patra, 2010). Likewise, the use of a purified PT extract from *A. nodosum* decreased gas production and feed degradability in rumen batch cultures when used at 0.1–0.5 g/L (Wang et al., 2008). In a previous dose response study using batch cultures, we also observed that ASC decreased gas production rate and methane emissions when used at 2 g/L but not with lower concentrations (Belanche et al., 2015a). In the present study ASC decreased neither OM, NDF, or ADF degradability, nor gas production in comparison to CON, possibly because of the slightly lower concentration used (1.56 g/L) or the greater dilution of the rumen fluid. Thus, our results suggest that PT from ASC had a similar ability to terrestrial tannins in the formation of protein-phenol complexes; however their ability to form carbohydrate-phenol complexes and to decrease rumen methanogenesis seemed to be less obvious.

*Laminaria digitata* (LAM) is a brown seaweed which grows in subtidal zones in cold-temperate waters. LAM had a higher content of N, ash and fucoxanthin than ASC but much lower PT content. The results described here showed that LAM had no effect on feed degradability, gas or methane emissions in comparison to CON diet. Similarly, a lack of effect of LAM on gas production and methanogenesis was reported in batch cultures at 2 g/L (Belanche et al., 2015a). This suggests the PT content in LAM is not high enough to have detrimental effects on feed degradability when incubated at 5% inclusion rate.

### Rumen fermentation

The mechanisms by which tannins reduce ruminal degradation of different dietary components are not entirely clear. Among the most accepted mechanisms are substrate privation (McMahon et al., 2000), enzyme inhibition (Jones et al., 1994) and direct inhibition of certain rumen microorganism (Scalbert, 1991). With respect to the first mechanism, the formation of complexes with proteins and carbohydrates render these nutrients inaccessible to microorganism limiting the necessary attachment of microorganisms for degradation to occur (McAllister et al., 1994). Our experiment demonstrated that supplementation of CON diet with brown seaweeds did not change the ruminal concentration of total bacteria nor the main fermentation products. This observation is in line with a meta-analysis which reported a small impact of terrestrial tannins on the VFA production (Patra, 2010). Thus, 5% inclusion of brown seaweed in the diet does not seem to compromise rumen microbial function.

With respect to enzyme inhibition, tannins are soluble and can react with microbial extracellular enzymes inhibiting their activity (McSweeney et al., 2001). Inhibition of α-glycosidase and α-amylase has been reported in rats fed PT resulting in a delay in the digestion of oligosaccharides and disaccharides (Li et al., 2011). In a batch culture using rumen fluid, it was observed that pure PT extract inhibited fiber degradation to a greater extent than the starch degradation (Wang et al., 2008). The data reported here mostly agree with this observation since ASC decreased the absolute carboxy-methyl cellulase activity (−37%) in comparison to CON, with no effect on amylase or xylanase activity (in terms of μmol of sugar released/ gDM·min). The effect of ASC on carboxy-methyl cellulase activity disappeared when it was expressed relative to the soluble protein concentration, which represents the enzymes which are not associated with PT. This observation suggests that formation of PT-enzyme complexes maybe the main factor which limits cellulase activity in ASC diets, and ultimately fiber degradability (Wang et al., 2008). On the contrary, LAM did not modify the absolute enzymatic activities in respect to CON diet, but increased xylanase (+70%) and carboxy-methyl-cellulase relative activity (+41%). The main reason for that was the lower soluble protein concentration observed in LAM than in CON diets (−19%). This suggests that enzyme inhibition is unlikely to be an important factor in LAM diets since a lower concentration of total enzymes but with a greater xylanase and carboxy-methyl-cellulase activity per unit of enzyme was observed in comparison to CON and ASC diets.

Regarding the possibility of direct inhibition of certain rumen microbes by tannins; an *in vivo* experiment in which steers and lambs were fed with increasing doses of commercial *A. nodosum* meal (Tasco-14™) revealed a decrease in the prevalence of *E. coli* O157:H7 in ruminant's feces with no further improvements in animal performance (Bach et al., 2008). Moreover, Wang et al. (2009) demonstrated *in vitro* that purified PT from *A. nodosum* inhibited growth of *E. coli* O157:H7 to a greater extent than commonly used terrestrial tannins. Our experiment, in which a non-pathogenic *E. coli* 0157:H7 strand was used, agrees with these observations since the post-inoculation numbers of *E. coli* showed a faster decay pattern in vessels fed ASC than those fed CON or LAM. Therefore, the use of ASC as feed ingredient seems to have certain anti-microbial properties in the rumen (see below) and it is able to decrease the concentration of *E. coli* which is currently considered an important fecal contaminator of carcasses and ultimately a source of foodborne disease in humans (Bach et al., 2008).

### Rumen microbial community

The observed decrease in N degradability with ASC (−24%) and LAM (−6%) resulted in a decreased concentration of soluble proteins (−14 and −20%) and ultimately in the concentration of methanogens (–1.06 and –0.60 log) and protozoa numbers (–2.84 and –2.72 log, respectively) with no impact on the concentration of bacteria or anaerobic fungi. Similar decreases in protozoa and methanogens numbers were observed in dairy cows fed diets with a shortage in rumen fermentable protein (Belanche et al., 2012) suggesting that methanogens and protozoa are particularly sensitive to the lack of fermentable protein in the rumen. In a dose response experiment in which the protozoal activity was tested according to their ability to predate ^14^C-labeleld bacteria, a linear decrease in protozoal activity when ASC was incubated up to 2 g/L was detected while no effect was observed using LAM (Belanche et al., 2015a). Thus, our results suggest that substrate privation is not the only mechanism by which seaweed modifies rumen function, since ASC and LAM had similar impact on the numbers of rumen microbes despite their dissimilar PT content.

Orpin et al. (1985) studied the rumen microbiota of Orkney sheep (North Ronaldsay, Scotland), of which the males survive almost entirely on a seaweed diet composed mainly of *Lamminaira, Fucus*, and *Ascophillum* species. It was noted that seaweed-eating sheep had a rumen microbial community with important differences in the structure and metabolic function compared to grass-fed sheep due to a microbial adaptation toward seaweed feeding. Our experiment demonstrated that the impact of seaweed on the rumen microbial community differs according to seaweed species: inclusion of 5% ASC in the diet promoted a moderate change in the structure of the bacterial community in comparison to the CON diet, while no differences were noted between LAM and CON diets. Although the antimicrobial mode of action of brown seaweeds is yet to be fully elucidated, the most feasible hypothesis is the formation of PT-protein complexes in the cell wall which limit the excretion of extracellular enzymes and the absorption of nutrients, as described for terrestrial tannins (McSweeney et al., 2001). This hypothesis was supported by the CCA data which showed that concentrations of soluble protein and branched chain fatty acids derived from protein degradation were positively correlated with the structure of bacterial community in vessels fed CON and LAM. These substrates could act as microbial growth factors under such dietary conditions, but if they are sequestrated in ASC diets this could alter the structure of the microbial community. In addition, ASC also has the highest content of polysaccharides among brown algae (42–70% in DM) some of which are bioactive such as alginic acid, fucoidan, laminarin, and mannitol (Holdt and Kraan, 2011). Thus, the observed shift in the bacterial community in vessels fed ASC could be due to the presence of these polysaccharides, most of which are indigestible and have functional properties as prebiotics for human and animals (O'Sullivan et al., 2010).

Incorporation of brown seaweeds into the diet neither decreased the concentration of total bacteria nor their diversity suggesting a similar bacterial community in vessels fed LAM and CON diets. This study revealed that ASC and LAM increased the levels of *Ruminobacter* (+1.41 and +1.20 log units, respectively), an amylolyic bacteria able to degrade proteins. Moreover, within the phylum *Firmicutes*; ASC promoted the highest concentration of *Pseudobutyrivibrio* (+0.26 log) while LAM increased the levels of *Streptococcus* (+1.25 log) compared to CON diets. *Streptococcus bovis*, which is known for rapid growth on starch or sugar, had an increased growth rate (from 0.9 to 1.6/h) when ammonia was replaced by AA in the media (Russell, 1993) suggesting that the higher protein concentration observed in vessels fed LAM could enhance the growth of this particular bacterium. This observation is in line with the greater abundance of *S. bovis* observed in seaweed fed Orkney sheep (Orpin et al., 1985); however this author also reported lower levels of cellulolytic bacteria in sheep fed seaweed, which was not observed in our experiment. Previous studies have shown that the growth of proteolytic bacteria (*Butyrivibrio fibrisolvens, Ruminobacter amylophylus* and *S. bovis*) was reduced by condensed tannins (Jones et al., 1994), while *Prevotella ruminicola, Clostridium* sp., *Streptococcus gallolyticus* and *Streptococcus caprinus* can tolerate high concentrations of tannins (Brooker et al., 1994; Nelson et al., 1998). Similarly, ruminal concentration of *Butyrivibrio proteoclasticum* was reduced, while *B. fibrisolvens* increased, in lambs supplemented with quebracho tannins promoting a shift in ruminal fat bio-hydrogenation pattern (Vasta et al., 2010). Thus, it appears from these studies that closely related bacterial strains of the same species can differ significantly in their tolerance to tannins, and here we demonstrate that this observation is also true for PT. The mechanism of tannin tolerance has not been elucidated but adaptive strategies based on the secretion of extracellular polysaccharide that separate the microbial cell wall from active tannins, and formation of a thick gycocalyx with high binding affinity to tannin have been described (McSweeney et al., 2001).

As expected, the methanogen community had a much lower diversity than observed for the bacterial community. This study also revealed that the structure and biodiversity of the methanogen community was unaffected by the use of brown seaweeds. Similarly, no effects of brown seaweed were observed in the distribution of the main protozoal groups. These observations suggest that brown seaweeds either have a no specific antimicrobial action against methanogens and protozoa; or that the dose used was not high enough to modify the structure of these microbial communities, which have previously been proven to be more stable than the bacterial community (Belanche et al., 2014, 2015b).

### Nitrogen metabolism and microbial protein synthesis

Because ASC is readily accessible, it is the main raw material for most seaweed meals and the large majority of experimental work to measure the effectiveness of seaweed meal has been carried out with this seaweed. This study revealed that ASC had not only a negative impact on N degradability (−24%) but also tended to lower the NAN overflow (−8.7%) and microbial protein synthesis (−6.9%) due to limited N availability for the rumen microbes. Adding ASC meal into the diet did not benefit animal performance in poultry and pigs (Indergaard and Minsaas, 1991). Nevertheless, several trials have shown beneficial effects using ASC in ruminants: including a 6.8% increase in milk production when cows were supplemented 200 g/d an *A. nodosum* meal (Jensen et al., 1968), whilst sheep fed 35 g/d seaweed meal over a 2-year period maintained their body weight more effectively during the winter and had 20% greater wool production than non-supplemented sheep (Saeter and Jensen, 1957). *A. nodosum* however is not very palatable and a decrease in DM intake was reported in Holstein calves fed 60 g/d *A. nodosum* meal (Erickson et al., 2012), therefore this seaweed is usually used in low concentrations, typically less than 5% of the diet (Makkar et al., 2016). These results seem to indicate that the negative impact of ASC on rumen protein degradability can be compensated by an increased flow of by-pass protein which can then be digested in the intestine due to the pH dependent nature of the protein-phenol complexes (Min et al., 2003).

However, dietary supplementation with brown seaweed does not always have a negative impact on the N metabolism since this effect depends on the seaweed species considered. Addition of 5% of LAM into conventional diets (CON) did not have detrimental effects on N metabolism and tended to increase outflows of NAN (+3.0%) from vessels and microbial-N (+7.6%) resulting in a substantial improvement in the efficiency of microbial protein synthesis per unit of degradable OM (+9.9%). The reason for this improvement is not fully understood but could rely on the adaptation of the rumen microbial ecosystem to tolerate small concentrations of PT. This adaptation could explain the preference of seaweed-eating sheep from North Ronaldsay for *Lamminaira* species rather than for other available seaweeds, such as, *Fucus, Ascophylum, Palmaria*, or *Alaria* (Orpin et al., 1985). Thus, more research is needed to determine the maximum tolerable dose of PT by the rumen microbes and by the animal.

## Conclusions

Here we show that a multi-omics approach based on a characterization of the rumen microbial communities together with an integrated description of the rumen fermentation process is necessary to understand the effect and mode of action of alternative feeds. It was noted *in vitro* that the effect of seaweed on the rumen function differed depending on the seaweed considered: ASC promoted a change in the structure of the bacterial community and had a negative impact on the N utilization as a result of its high PT content. However, LAM which has a much lower PT content did not have detrimental effects on N degradability and increased the xylanase and carboxy-methyl-cellulose enzymatic activity resulting in a greater efficiency of microbial protein synthesis in comparison to CON diets. Further studies are required *in vivo* to determine the optimum and/or the maximum seaweed inclusion rate into the diet which does not impair the rumen function based on a similar multi-omics approach to that described in this paper.

## Author contributions

AB and CN designed the experiment; AB, EJ, and IP conducted the research; AB wrote the manuscript; AB, EJ, IP, and CN reviewed the manuscript. AB had primary responsibility for the final content. All authors read and approved the final manuscript.

### Conflict of interest statement

The authors declare that the research was conducted in the absence of any commercial or financial relationships that could be construed as a potential conflict of interest.
