# Supplementary material for: A Metagenomics Approach to Evaluate the Impact of Dietary Supplementation with Ascophyllum nodosum or Laminaria digitata on Rumen Function in Rusitec Fermenters
Source: Front Microbiol. 2016 Mar 10;7:299. doi: 10.3389/fmicb.2016.00299 (PMC4785176; doi:10.3389/fmicb.2016.00299)
Supplement: Supplementary file 1 [file Table1.DOCX]

**ONLINE SUPPORTING MATERIAL**

**Supplemental Table 1**. Ingredients and chemical composition of the experimental diets.

| **Diets^1^** | **CON** | **ASC** | **LAM** |
| --- | --- | --- | --- |
| **Ingredients (g/kg)** |  |  |  |
| Alfalfa hay | 300 | 285 | 285 |
| Grass hay | 200 | 190 | 190 |
| Barley | 300 | 285 | 285 |
| Corn | 120 | 114 | 114 |
| Soya bean meal | 77 | 73 | 73 |
| Brown seaweed | 0 | 50 | 50 |
| Vitamin premix^1^ | 3 | 3 | 3 |
| **Composition (g/kg)** | |  |  |
| Organic matter | 949 | 940 | 937 |
| Nitrogen | 20.5 | 20.4 | 20.8 |
| Neutral detergent fibre | 370 | 366 | 371 |
| Acid detergent fibre | 211 | 212 | 214 |

^1^ Rumins Cattle GP (Rumeco Ltd, UK.) Declared composition: Ca 240, P 20, Mg 50, Na 80, Se 0.03, Co 0.09, I 0.4, Mn 3, Zn 4 and Cu 1.5 g/kg, retinol 4×10^5^, cholecalciferol 8×10^4^ and α-tocopherol 10^3^ IU/kg

**Supplemental Table 2**. Primers used for quantitative PCR and Ion-Torrent Next Generation Sequencing.

| **Target** | **Author** | **Forward Primer** | **Reverse Primer** | **T^a^** | | **Amplicon (bp)** |
| --- | --- | --- | --- | --- | --- | --- |
| Quantitative PCR |  |  |  | |  |  |
| Total bacteria | ([Maeda*, et al.*, 2003](#_ENREF_24)) | GTGSTGCAYGGYTGTCGTCA | ACGTCRTCCMCACCTTCCTC | | 61 | 150 |
| Total protozoa | ([Sylvester*, et al.*, 2004](#_ENREF_48)) | GCTTTCGWTGGTAGTGTATT | CTTGCCCTCYAATCGTWCT | | 55 | 223 |
| Anaerobic fungi | ([Denman & McSweeney, 2006](#_ENREF_14)) | GAGGAAGTAAAAGTCGTAACAAGGTTTC | CAAATTCACAAAGGGTAGGATGATT | | 62 | 120 |
| Methanogens | ([Denman*, et al.*, 2007](#_ENREF_15)) | TTCGGTGGATCDCARAGRGC | GBARGTCGWAWCCGTAGAATCC | | 56 | 140 |
| Ion Torrent NGS |  |  |  | |  |  |
| Bacterial primers | ([Spear*, et al.*, 2008](#_ENREF_44)) | AGAGTTTGATCMTGGCTCAG | CTGCTGCCTYCCGTA | | 58 | 348 |
| Bacterial Adaptors |  | CCATCTCATCCCTGCGTGTCTCCGACTCAG | CCTCTCTATGGGCAGTCGGTGAT | |  |  |
| Methanogens primers | ([Wright & Pimm, 2003](#_ENREF_55)) | GCTCAGTAACACGTGG | GWATTACCGCGGCKGCTG | | 58 | 433 |
| Methanogens adaptors |  | CCATCTCATCCCTGCGTGTCTCCGACTCAG | CCTCTCTATGGGCAGTCGGTGAT | |  |  |

**Supplemental Table 3.** Effect of supplementing a control diet (CON) with *Ascophyllum nodosum* (ASC) and *Laminaria digitata* (LAM) on the relative abundance of the main bacteria at phylum, family and genus level in a Rusitec system. The total number of reads per sample was log-transformed and minor genera were discarded. Within a raw means without a common superscript differ (*P* < 0.05).

| **Phylum** | **Family** | **Genus** | **CON** | **ASC** | **LAM** | **SED^1^** | ***P*-value** |
| --- | --- | --- | --- | --- | --- | --- | --- |
| *Bacteroidetes* |  |  | 4.02 | 4.07 | 4.04 | 0.052 | 0.664 |
|  | *Flammeovirgaceae* |  | 2.64^a^ | 2.59^a^ | 1.76^b^ | 0.195 | 0.007 |
|  |  | *Sediminitomix* | 1.01 | 1.52 | 1.01 | 0.381 | 0.364 |
|  | *Prevotellaceae* |  | 3.94 | 3.95 | 3.96 | 0.081 | 0.984 |
|  |  | *Paraprevotella* | 1.81 | 1.73 | 1.81 | 0.163 | 0.849 |
|  |  | *Prevotella* | 3.92 | 3.93 | 3.94 | 0.085 | 0.981 |
|  | *Rikenellaceae* | *Rikenella* | 1.33 | 1.24 | 1.22 | 0.071 | 0.319 |
| *Fibrobacteres* | *Fibrobacteraceae* | *Fibrobacter* | 2.36 | 2.34 | 2.30 | 0.294 | 0.976 |
| *Firmicutes* |  |  | 3.78 | 3.70 | 3.69 | 0.045 | 0.182 |
|  | *Acidaminococcaceae* | | 2.87 | 2.70 | 2.76 | 0.175 | 0.636 |
|  |  | *Acidaminococcus* | 2.18 | 2.12 | 2.33 | 0.172 | 0.489 |
|  |  | *Succiniclasticum* | 2.74 | 2.56 | 2.51 | 0.227 | 0.590 |
|  | *Clostridiaceae* | *Clostridium sensu stricto* | 0.83 | 1.41 | 1.27 | 0.324 | 0.250 |
|  | *Clostridiales_Incertae Sedis XIII* | | 0.97 | 1.51 | 0.89 | 0.234 | 0.073 |
|  | *Erysipelotrichaceae* | | 2.18 | 2.21 | 2.39 | 0.251 | 0.691 |
|  |  | *Solobacterium* | 2.12 | 1.90 | 2.01 | 0.261 | 0.703 |
|  | *Lachnospiraceae* |  | 2.64 | 2.74 | 2.77 | 0.124 | 0.599 |
|  |  | *Butyrivibrio* | 1.64 | 1.73 | 1.61 | 0.150 | 0.735 |
|  |  | *Oribacterium* | 1.64 | 1.50 | 1.49 | 0.141 | 0.518 |
|  |  | *Pseudobutyrivibrio* | 0.48^ab^ | 0.74^a^ | 0.42^b^ | 0.103 | 0.045 |
|  |  | *Roseburia* | 1.82 | 1.69 | 1.87 | 0.325 | 0.851 |
|  | *Lactobacillaceae* | *Lactobacillus* | 2.20 | 2.35 | 2.45 | 0.252 | 0.636 |
|  | *Ruminococcaceae* |  | 1.81 | 2.11 | 1.89 | 0.156 | 0.212 |
|  |  | *Ruminococcus* | 1.03 | 1.30 | 1.39 | 0.269 | 0.426 |
|  | *Streptococcaceae* | *Streptococcus* | 1.36^b^ | 2.01^ab^ | 2.61^a^ | 0.353 | 0.034 |
|  | *Veillonellaceae* |  | 2.18 | 2.26 | 2.19 | 0.064 | 0.449 |
|  |  | *Anaerovibrio* | 1.38 | 1.37 | 1.42 | 0.132 | 0.909 |
|  |  | *Megasphaera* | 0.73 | 0.60 | 0.92 | 0.201 | 0.357 |
|  |  | *Mitsuokella* | 1.25 | 1.35 | 1.32 | 0.209 | 0.885 |
|  |  | *Schwartzia* | 1.76 | 1.89 | 1.80 | 0.047 | 0.067 |
|  |  | *Selenomonas* | 1.56 | 1.58 | 1.41 | 0.115 | 0.349 |
| *Proteobacteria* |  |  | 2.83 | 3.02 | 3.14 | 0.101 | 0.061 |
|  | *Succinivibrionaceae* | | 2.76 | 2.94 | 3.09 | 0.107 | 0.058 |
|  |  | *Ruminobacter* | 0.95^b^ | 2.36^a^ | 2.15^a^ | 0.373 | 0.019 |
|  |  | *Succinivibrio* | 2.73 | 2.74 | 2.92 | 0.110 | 0.249 |
|  | *Sutterellaceae* | *Parasutterella* | 1.16 | 1.30 | 1.29 | 0.160 | 0.632 |
| *Spirochaetes* | *Spirochaetaceae* | *Treponema* | 2.67 | 2.81 | 2.65 | 0.093 | 0.272 |
| *Tenericutes* |  |  | 2.78 | 2.86 | 2.84 | 0.097 | 0.701 |
|  | *Anaeroplasmataceae* | | 2.78 | 2.86 | 2.84 | 0.097 | 0.703 |
|  |  | *Anaeroplasma* | 2.74 | 2.82 | 2.75 | 0.142 | 0.844 |
|  |  | *Asteroleplasma* | 1.60 | 1.66 | 1.76 | 0.260 | 0.824 |
| *Unclassified* |  |  | 3.75 | 3.63 | 3.73 | 0.107 | 0.506 |

**Suplemental Table 4.** Effect of supplementing a control diet (CON) with *Ascophyllum nodosum* (ASC) and *Laminaria digitata* (LAM) on the relative abundance of the main archaea at family, genus and species level in the Rusitec system. The total number of reads per sample was log-transformed.

| **Family** | **Genus** | **Species** | **CON** | **ASC** | **LAM** | **SED^1^** | ***P*-value** |
| --- | --- | --- | --- | --- | --- | --- | --- |
|  |  |  |  |  |  |  |  |
| *Methanomassiliicoccaceae* | | | 2.99 | 2.99 | 2.99 | 0.006 | 0.465 |
|  | *Group 11* |  | 2.88 | 2.45 | 2.74 | 0.232 | 0.245 |
|  |  | *Methanomethylophilus alvus* | 2.88 | 2.37 | 2.72 | 0.284 | 0.264 |
|  |  | *Others* | 0.95 | 1.12 | 1.00 | 0.236 | 0.753 |
|  | *Group 12* |  | 1.91 | 2.64 | 2.37 | 0.418 | 0.290 |
|  | *Group 3a* |  | 0.99 | 1.16 | 0.95 | 0.248 | 0.670 |
|  | *Group 9* |  | 0.95 | 1.15 | 1.05 | 0.358 | 0.856 |
| *Methanobacteriaceae* | | | 1.06 | 1.09 | 1.11 | 0.239 | 0.971 |
|  | *Methanobrevibacter* | | 1.06 | 1.09 | 1.11 | 0.239 | 0.971 |
|  |  | *M bovis koreani* | 0.49 | 0.31 | 0.50 | 0.398 | 0.872 |
|  |  | *M gottschalkii* | 0.85 | 1.04 | 0.98 | 0.264 | 0.787 |
|  |  | *M wolinii* | 0.12 | 0.00 | 0.25 | 0.240 | 0.608 |
